# Clinical Analysis of Postpartum Preeclampsia After an Uncomplicated Pregnancy

**DOI:** 10.7759/cureus.57834

**Published:** 2024-04-08

**Authors:** Shannon L Fehr, Steve Frelier

**Affiliations:** 1 Anatomical Sciences, Liberty University College of Osteopathic Medicine, Lynchburg, USA; 2 Internal Medicine, Southside Regional Medical Center, Petersburg, USA

**Keywords:** postpartum preeclampsia, postpartum dyspnea, postpartum pulmonary edema, postpartum respiratory distress, medical disorders in pregnancy, uncomplicated term pregnancies, preeclampsia with severe features, emergency medical service

## Abstract

Preeclampsia is a rare complication of pregnancy and can cause maternal death. This case report serves to increase awareness of the range and severity of symptoms in postpartum preeclampsia and highlights a stepwise approach to provide prompt management. The cause of preeclampsia is not fully understood but is correlated with many placental and maternal factors. Preeclampsia typically resolves with delivery, and it is uncommon to have symptoms in the postpartum period. Due to the rarity of the disease, it is not typically at the top of a differential list for postpartum women. A 35-year-old first-time mother presents with shortness of breath, tightness in her chest, and mild pulmonary edema. A series of chest X-rays, computed tomographic angiogram of the chest, and serial labs reveal she has postpartum preeclampsia. Many pre-eclamptic symptoms are common to a variety of conditions leading to an extensive list of differential diagnoses. Used in a stepwise fashion, clinical analysis allows physicians to accurately diagnose postpartum preeclampsia and provide lifesaving treatment for these mothers. This study highlights the need for vigilance in symptom analysis and diagnostic testing.

## Introduction

Preeclampsia is a condition with a complex pathogenesis of placental and maternal factors affecting 5-7% of pregnant women worldwide [1]. The disease is defined as new-onset hypertension and proteinuria or new-onset hypertension and end-organ failure of one or more organ systems [2,3]. Diagnostic criteria consist of systolic blood pressure greater than 140 mmHg and/or diastolic blood pressure greater than 90 mmHg and proteinuria greater than 0.3 g/24h in a urine specimen. End organ dysfunction can include but is not limited: to thrombocytopenia less than 100,000, liver transaminases twice the upper limit, visual symptoms, uteroplacental dysfunction, acute kidney injury, pulmonary edema, and new onset headache. Severe preeclampsia is defined as systolic blood pressure greater than 160 mmHg and significant end-organ damage [2]. Risk factors include prior history of preeclampsia, chronic hypertension, pregestational diabetes, systemic lupus erythematous, antiphospholipid syndrome, obesity, advanced maternal age, and nulliparity. Research studies suggest the pathophysiology of failed spiral artery remodeling leads to hypoxia and oxidative stress and the release of antiangiogenic factors [2]. This results in maternal endothelial dysfunction and widespread clinical manifestations. Physicians are keen to diagnose preeclampsia because it is one of the four leading causes of maternal death and can cause oligohydramnios and growth restriction to the fetus. Preeclampsia is diagnosed in the second half of pregnancy, and most commonly develops after 34 weeks [1,3]. There is a gap in research on the incidence of new-onset postpartum preeclampsia and this case report will highlight the need for further studies.

## Case presentation

Initial presentation and imaging

**Figure 1:**
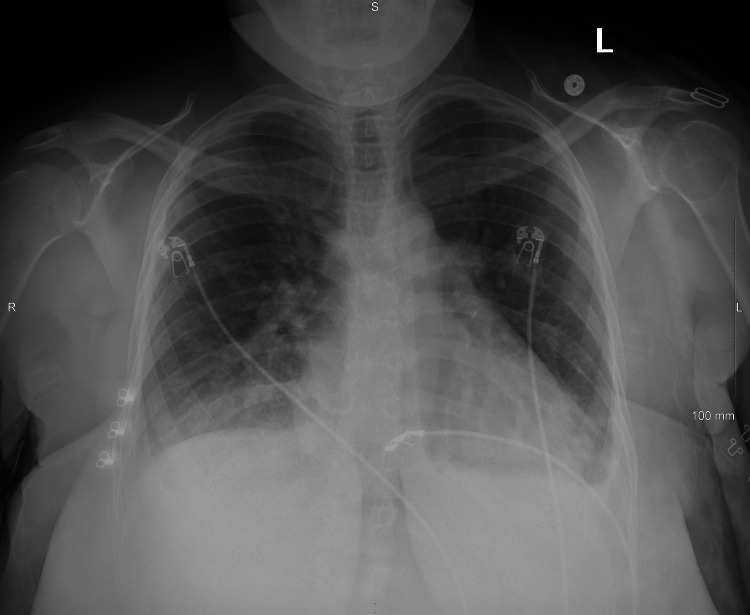
Chest X-ray demonstrating mild pulmonary edema with minimal bilateral pleural effusion

A 35-year-old female with a BMI of 43.2 kg/m^2^ and a past medical history of bradycardia, anxiety, depression, and Herpes simplex virus-2 presented to the ER one week post-partum with a chief complaint of shortness of breath. She also described the feeling of a bubble in the middle of her chest. Associated symptoms were positive for bilateral lower extremity edema. Her pregnancy and vaginal delivery were uncomplicated and there was no family history of cardiac disease. Vital signs on admission revealed a heart rate of 48, blood pressure of 170/90 mmHg, respiration rate of 38 breaths/minute, temperature of 98.9 degrees Fahrenheit, and an oxygen saturation (SpO_2_) of 97%. This patient appeared to be out of breath and in an anxious state. Auscultation of the heart and lungs revealed normal S1 and S2 with clear lung sounds in all fields. Cranial nerves II-XII were intact. Bilateral lower extremity edema was identified without pitting. An initial electrocardiogram demonstrated bradycardia with a regular rhythm and no ST changes. A chest X-ray demonstrated mild pulmonary edema with small bilateral pleural effusions (Figure 1).

**Figure 2:**
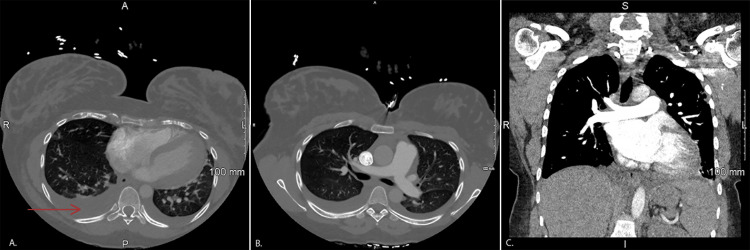
CT chest demonstrating mild bilateral effusions and negative pulmonary embolism A. Lower lung field plain with an arrow indicating pleural effusion present bilaterally. R: Right, L: left, A: Anterior, P: Posterior B. Thoracic CT with clear main pulmonary artery indicating negative saddle embolus. C. Coronal CT angiogram chest example of clear pulmonary arteries indicating negative pulmonary embolism. S: Superior; I: Inferior

An echocardiogram (ECHO) showed normal ventricular function with an estimated ejection fraction of 60-65% and was significant only for moderate mitral regurgitation. Ventricular and atrial chamber measurements were within normal limits. Her complete blood count (CBC) revealed normocytic anemia and her complete metabolic panel revealed an elevated N-terminal pro-B-type natriuretic peptide (NT-proBNP), alkaline phosphatase, alanine transaminase, and aspartate transaminase (Table 1). Urinalysis (UA) demonstrated turbidity with the presence of elevated protein, red blood cells, and white blood cells without the presence of bacteria (Table 2). It was also found that cholesterol and D-dimer levels were elevated (Table 4). Computed tomographic angiogram (CTA) was negative for pulmonary embolism (PE) but again demonstrated mild pulmonary edema and bilateral pleural effusions (Figure 2).

**Table 1:** Complete metabolic panel and cardiac panel Combined laboratory values for a complete metabolic panel, lipid profile, and cardiac markers. BUN: Blood urea nitrogen; GFR: Glomerular filtration rate; HDL: High-density lipoprotein; LDL: Low-density lipoprotein; VLDL: Very low-density lipoprotein; NT-proBNP: N-terminal prohormone of brain natriuretic peptide; ALT: Alanine transaminase; AST: Aspartate transaminase

Chemistry	Value	Reference range
Sodium	145	13-145 mmol/L
Potassium	3.9	3.7-5.2 mmol/L
Chloride	112	96-106 mmol/L
CO_2_	26	23-29 mmol/L
BUN	18	6-20 mg/dL
Creatinine	0.72	0.6-1.3 mg/dL
BUN/Cr ratio	11	10-20
Anion gap	7	4-12 mmol/L
Est. GFR	>60	>60 mL/min/1.73m^2^
Glucose, random	74	70-100 mg/dL
Calcium	8.7	8.5-10.2 mg/dL
Total protein	6	6.0-8.3 g/dL
Albumin	3.9	3.4-5.4 g/dL
NT-proBNP	1220	<125 pg/mL
Alkaline phosphatase	125	20-135 U/L
ALT	132	4-36 U/L
AST	79	8-33 U/L
Bilirubin (total)	0.4	0.1-1.2 mg/dL
Troponin high sensitivity	9	<14 ng/L
Cholesterol/HDL ratio	3.5	1-3.5
Cholesterol (total)	205	125-200 mg/dL
HDL cholesterol	58	40-60 mg/dL
LDL cholesterol	124.2	<100 mg/dL
Triglycerides	114	<150 mg/dL
VLDL-cholesterol calculated	22.8	2-30 mg/dL
D-dimer quant	3.78	<0.5

**Table 2:** Urinalysis WBC: White blood cells; RBC: Red blood cells; hpf: High power field

Chemistries	Value	Reference range
Color	Yellow/turbid	Clear/pale yellow-dark amber
Glucose	Negative	Negative
Bilirubin	Negative	Negative
Ketones	Negative	Negative
Specific gravity	1.009	1.002-1.030
Blood	Large	Negative
pH	7.0	5-9
Protein	42	0-14 mg/dL
Urobilinogen	0.1	<1 mg/dL
Nitrite	Negative	Negative
Leukocyte esterase	Large	Negative
Appearance	Turbid	Clear
Mucus	Trace	Negative
WBC	>100	0-4/hpf
RBC	>100	0-3/hpf
Epithelial cells	Many	0-2/hpf
Bacteria	Negative	Negative
Creatinine	31.0	0-150mg

Timeline to a diagnostic conclusion

The process of diagnosing this patient demonstrated the importance of a stepwise approach to correctly identify the diagnosis. The sequence of diagnostic test results directed subsequent tests for this patient. It initially appeared that she was experiencing postpartum cardiomyopathy and fluid overload due to her shortness of breath, elevated NT-proBNP, and lower extremity edema. Intravenous (IV) diuretics were started to reduce pulmonary edema and lower extremity edema and hydralazine was given to rapidly reduce her blood pressure. An ECHO was ordered to confirm this diagnosis. Because her ECHO demonstrated normal systolic function and the pulmonary edema was not extensive, this was not a convincing case of fluid overload heart failure. She continued to have an increased work of breathing and required multiple breaks in history telling. The next step was to order a CTA and start heparin. Although she had no pain or indication of a deep venous thrombosis, she did have tachypnea, chest tightness, and an elevated D-dimer. She had a Wells score of 4.5 indicating a moderate risk for PE. CTA would identify a PE if present and can confirm the extent of the pulmonary edema. Heparin was discontinued because the CTA was negative for PE (Figures 2B, 2C). Although the X-ray and CT did show bilateral pleural effusions, her clinical presentation was more extensive than the radiological findings (Figures 1, 2A). This indicated there was a further underlying cause of the pleural effusions leading to her shortness of breath. Subsequently, CBC and UA results were analyzed and significant for elevated liver enzymes, proteinuria, and hematuria. This step required clinical reasoning given symptoms that were not caused by typical diagnoses. The diagnosis was then determined using the laboratory results of severe proteinuria, elevated liver enzymes, hypertensive urgency, and pulmonary edema to be severe postpartum onset preeclampsia. She was admitted to the intensive care unit (ICU) for observation, and she was diagnosed with postpartum preeclampsia with severe features. Stat IV magnesium was started for the next 24 hours. She was also started on lisinopril and metoprolol while vitals were closely monitored. After one day in the ICU, she remained mildly short of breath and her blood pressure had decreased. Serial labs revealed her liver transaminases were decreasing and her chest x-ray showed improvement in her pulmonary edema. The morning of her third day in the hospital, she was feeling better, and her labs were back to their baseline. Vitals were stable on these medications with a heart rate of 73 beats per minute, blood pressure of 122/70 mmHg, and respirations of 16 breaths/minute. She was counseled on the importance of maintaining furosemide, lisinopril, and metoprolol to keep tight control of her blood pressure. She was functionally able to ambulate without shortness of breath, the swelling had decreased in her legs, and she demonstrated overall clinical improvement.

## Discussion

Preeclampsia is a disorder presenting in the second half of pregnancy and less commonly during the first six weeks of the postpartum period [1]. It is treated with 24 hours of IV magnesium sulfate and antihypertensive therapy is used to reduce hypertensive urgency and prevent stroke. Intrapartum care includes frequent monitoring of blood pressure, pulse oximetry, imaging studies as well as labs such as NT-proBNP [2].

All women are monitored for these features at prenatal visits allowing physicians to promptly address preeclamptic symptoms to protect mom and baby. When there are no intrapartum symptoms or complications, it is more difficult to diagnose as preeclampsia is often thought of as a condition of pregnancy. In the setting of postpartum onset, a stepwise approach to rule out other common conditions is important. About 60-70% of women with postpartum onset present with neurologic symptoms, which calls for stat imaging and neurologic consultation to rule out cerebrovascular entities [2,4]. Because postpartum blood pressure monitoring is not protocol for women without preceding blood pressure concerns, any incidental finding or finding alongside another symptom is an indication to investigate for preeclampsia. As in this case, early symptoms are not always conducive to a single diagnosis. Blood was drawn on arrival to begin laboratory studies. After a thorough history and physical, a chest X-ray was completed. This step aims to correlate subjective and objective symptoms. The clinical picture of edema and shortness of breath points to cardiomyopathy and the next appropriate step was to perform an ECHO with contrast, bubble, strain, and 3D visualization. This adequately ruled out cardiomyopathy as the cause of acute heart failure because there was no systolic dysfunction.

These results may not have appeared directive at first, but with further tests, physicians were able to understand that her symptoms of apparent heart failure were in fact due to underlying preeclampsia. The ECHO showing mitral regurgitation likely represents secondary or functional mitral insufficiency, as there is no prior indication of cardiac dysfunction. The pre-eclamptic dysfunction led to fluid overload; her pulmonary vasculature had increased hydrostatic pressure and decreased oncotic pressure leading to edema [4,5]. This causes this patient’s presentation of acute decompensated heart failure. The increased left ventricular diastolic volume or preload dilates the mitral valve annulus and laterally displaces the papillary muscles resulting in taut stretching of the chordae tendineae [6]. Thus, her intrinsically normal mitral valve resulted in insufficient closure due to the combination of a dilated mitral annulus and restricted motion of the chordae tendineae.

In using a stepwise analysis, PE must be ruled out next. This patient had a moderate risk for PE and CTA was correctly used to narrow the differential. After determining that her shortness of breath and acute heart failure symptoms were not caused by cardiomyopathy or PE, final labs were completed to accurately diagnose this patient. The presence of elevated liver transaminases alongside hypertensive urgency immediately led to the diagnosis of preeclampsia [7]. Typically, blood pressure must be elevated on two different occasions for the diagnosis. However, a systolic >160 mmHg is diagnostic within minutes, as in the case of this patient. UA also demonstrated proteinuria and hematuria without a urinary tract infection. This step confirmed the diagnosis and other presenting symptoms could now be understood as a product of this multisystem disorder. This case is classified as postpartum preeclampsia with severe features due to systolic blood pressures greater than 160 mmHg and the presence of end-organ damage including pulmonary edema. 

Although the exact pathophysiology of pre-eclampsia has yet to be determined, studies have directed attention to the abnormal remodeling of maternal spiral arteries. The physiologic process during pregnancy involves the loss of vascular endothelial and smooth muscle cells from spiral arteries and a replacement of fetal trophoblastic cells [8,9]. This allows a greater volume of blood into the intervillous space to provide sufficient nutrients to the growing fetus. The spiral arteries will continue to remodel during the first 22 weeks of pregnancy and will coincide with cytotrophoblast invasion after week 10. The largest area of invasion and the greatest increase in blood vessel diameter occurs in the center of the placenta. Studies have shown a 10-fold increase in diameter with a reduced pressure through the blood vessels [10]. Thus, failure or inadequate remodeling of the spiral arteries poses a risk to placental insufficiency. Oxidative stress and placental insufficiency can both lead to abnormal signaling from placental to maternal circulation and maternal pre-eclamptic syndrome. One of the main signaling theories involves syncytiotrophoblasts, which form the epithelial covering of the placental villi. This epithelial layer contacts the maternal blood, and when oxidative stress occurs, it will over-release cytokines, anti-angiogenic agents, pro-inflammatory factors, and other signaling molecules into the maternal blood. Some of these molecules include soluble fms-like tyrosine kinase-1 (sFlt-1) and soluble endoglin, which have been shown to decrease levels of vascular endothelial growth factor (VEGF) and transforming growth factor-β (TGF-β) [11]. This effectively decreases the growth and migration of endothelial cells creating a vasoconstrictive state. When these signaling molecules bind maternal cells, a systemic inflammatory response is created [5,12].

This systemic inflammatory response causes a variety of symptoms that vary between women due to genetic and acquired risk factors. The overflow of signaling molecules leads to the increased sympathetic response, renin-angiotensin-aldosterone system, and endothelin-1 creating systemic vasoconstriction. Once hypertension is present, other organ systems are more easily affected. Proteinuria occurs due to an increased renal tubular permeability. The sFlt-1 inhibition of VEGF also causes injury to glomerular endothelial cells [13,14]. Damaged podocytes and increased fenestrations allow larger protein molecules to be taken out of filtrate without reabsorption. The lack of protein in circulation also causes edema in many patients with severe features. Headaches in pre-eclamptic patients are also due to loss of fenestrations in the choroid plexus due to decreased VEGF and TGF-β. Increased filtration leads to periventricular edema and a variety of neurological symptoms [13]. Visual symptoms occur due to neurologic dysfunction, hypertensive retinopathy, and fluid accumulation behind the retina. Pulmonary edema and secondary mitral valve insufficiency may occur due to fluid overload states [14]. Increased fluid levels result in increased left diastolic volume and a dilated mitral annulus [5,6]. This can further result in lateral displacement of the papillary muscles and taut stretching of the chordae tendineae leading to a regurgitation of blood through the mitral valve.

Preeclampsia is typically resolved by the delivery of the child leading to the return of maternal endothelial function. There is a gap in knowledge and a lack of protocol for postpartum management, but management can cater to the remaining symptoms for each woman [8]. Intrapartum preeclamptic mothers will stay in the hospital longer after delivery to ensure a reduction in blood pressure and determine whether outpatient medications are required [2]. Research has shown that low-dose aspirin may prevent a recurrence of preeclampsia after a diagnosis has been made [4]. The setting of postpartum onset also calls for a 24-hour dose of magnesium sulfate and extended dosing may be considered contingent on clinical improvement. This patient showed improvement on the first day and this was not necessary. Hydralazine was used for a rapid reduction of blood pressure in this case due to hypertensive urgency. After clinical discussion, this patient was started on two blood pressure medications due to the extent of her symptoms. She will continue these medications as an outpatient to ensure a stable lowering occurs. There was a long discussion regarding follow-up with her primary care provider in the next 2-4 weeks to monitor her blood pressure. She will likely be able to discontinue usage when completely stable. Referral to a cardiologist was also discussed for follow-up of functional mitral insufficiency. She is at risk for preeclampsia in future pregnancies and the need for preeclamptic prophylaxis was explained [8].

## Conclusions

In conclusion, postpartum onset preeclampsia can be challenging to diagnose and treat in a timely manner. This case report serves to highlight symptoms and potential presentations of the uncommon postpartum onset preeclampsia. It is important to acknowledge that the complex etiology of preeclampsia does not exclude patients from the differential. Presenting symptoms often fall into the category of multiple diagnoses while lacking typical presentations of the underlying diagnosis, as in the case of this patient. One of the common presenting symptoms of hypertension was present on arrival of this patient and served as a vital and unique indicator to investigate preeclampsia. This highlights subtle indications to rule in potential postpartum in patients with broad-based symptoms and demonstrates the need for reputable steps in clinical analysis. Future studies should focus on refining postpartum protocols to prevent preeclamptic maternal morbidities.
